# Identifying areas of Australia with high out-of-hospital cardiac arrest incidence and low bystander cardiopulmonary resuscitation rates: A retrospective, observational study

**DOI:** 10.1371/journal.pone.0301176

**Published:** 2024-04-23

**Authors:** Tan Doan, Stuart Howell, Stephen Ball, Judith Finn, Peter Cameron, Emma Bosley, Bridget Dicker, Steven Faddy, Ziad Nehme, Natalie Heriot, Andy Swain, Melanie Thorrowgood, Andrew Thomas, Samuel Perillo, Mike McDermott, Tony Smith, Karen Smith, Jason Belcher, Janet Bray, on behalf of the Aus-ROC OHCA Epistry Management Committee

**Affiliations:** 1 Queensland Ambulance Service, Brisbane, Queensland, Australia; 2 School of Public Health and Preventive Medicine, Monash University, Clayton, Victoria, Australia; 3 Prehospital, Resuscitation and Emergency Care Research Unit (PRECRU), Curtin University, Bentley, Western Australia, Australia; 4 St John Western Australia, Belmont, Western Australia, Australia; 5 Emergency and Trauma Centre, The Alfred, Melbourne, Victoria, Australia; 6 School of Clinical Sciences, Queensland University of Technology, Brisbane City, Queensland, Australia; 7 Hato Hone St John New Zealand, Auckland, New Zealand; 8 Auckland University of Technology, Auckland, New Zealand; 9 NSW Ambulance, Sydney, New South Wales, Australia; 10 Ambulance Victoria, Doncaster, Victoria, Australia; 11 Wellington Free Ambulance, Wellington, New Zealand; 12 SA Ambulance Service, Adelaide, South Australia, Australia; 13 St John Ambulance NT, Darwin, Northern Territory, Australia; 14 ACT Ambulance, Canberra, Australian Capital Territory, Australia; 15 Ambulance Tasmania, Tasmania, Australia; 16 Research and Innovation, Silverchain, Victoria, Australia; Niigata University of Health and Welfare: Niigata Iryo Fukushi Daigaku, JAPAN

## Abstract

**Aim:**

This study aims to explore regional variation and identify regions within Australia with high incidence of out-of-hospital cardiac arrest (OHCA) and low rates of bystander cardiopulmonary resuscitation (CPR).

**Method:**

Adult OHCAs of presumed medical aetiology occurring across Australia between 2017 and 2019 were mapped onto local government areas (LGA) using the location of arrest coordinates. Bayesian spatial models were applied to provide “smoothed” estimates of OHCA incidence and bystander CPR rates (for bystander-witnessed OHCAs) for each LGA. For each state and territory, high-risk LGAs were defined as those with an incidence rate greater than the state or territory’s 75^th^ percentile and a bystander CPR rate less than the state or territory’s 25^th^ percentile.

**Results:**

A total of 62,579 OHCA cases attended by emergency medical services across 543 LGAs nationwide were included in the study. Nationally, the OHCA incidence rate across LGA ranged from 58.5 to 198.3 persons per 100,000, while bystander CPR rates ranged from 45% to 75%. We identified 60 high-risk LGAs, which were predominantly located in the state of New South Wales. Within each region, high-risk LGAs were typically located in regional and remote areas of the country, except for four metropolitan areas–two in Adelaide and two in Perth.

**Conclusions:**

We have identified high-risk LGAs, characterised by high incidence and low bystander CPR rates, which are predominantly in regional and remote areas of Australia. Strategies for reducing OHCA and improving bystander response may be best targeted at these regions.

## Introduction

Out-of-hospital cardiac arrest (OHCA) remains a significant public health problem globally, with incidence rates ranging from 30.0 to 97.1 individuals per 100,000 population across nations. Survival following OHCA is typically poor with survival to hospital discharge/30 days varying across countries from 3.1% to 24.0% [1]. In Australia, there are approximately 26,000 cases annually with only 12% of patients treated by emergency medical service (EMS) surviving to hospital discharge/30 days [2]. Survival depends on rapid implementation of a series of interventions known collectively as the Chain of Survival. Early bystander cardiopulmonary resuscitation (CPR) continues to be one of the most important of these interventions [3, 4].

A recent report has shown that OHCA incidence and bystander CPR rates vary considerably between Australian states and territories [2]. Crude incidence rates for all OHCAs attended by EMS ranged from 102.3 to 120.6 per 100,000 persons, while bystander CPR rates in EMS-treated OHCAs (bystander-witnessed and unwitnessed) varied between 61.3% and 78.2%. However, there is also emerging evidence of even more localised variation. Two studies have reported regional variations in OHCA incidence across smaller regions, local government areas (LGAs), within the Australian states of Victoria [5] and Queensland [6]. The Victorian study also reported regional variation in bystander CPR rates in bystander-witnessed OHCAs. However, the existence of such variation at the LGA level in other parts of Australia has not been explored.

Identifying regions at greatest risk, those with the highest incidence and lowest rates of bystander CPR, could facilitate the implementation of targeted intervention strategies. Therefore, this study aims to 1) examine bystander CPR rates and OHCA incidence by LGA across Australia and 2) identify high-risk LGAs at the national level as well as in each state and territory. Such information will help to identify appropriate public health interventions.

## Method

### Setting

This study was conducted in Australia, with a current population of approximately 26 million people. Australia is divided into 6 states (New South Wales [NSW], Victoria, Queensland, South Australia [SA], Western Australia [WA], Tasmania) and 2 territories (Australian Capital Territory [ACT], Northern Territory [NT]) (S1 Fig).

### Data source

This study used 2017–2019 data from the Australasian Resuscitation Outcomes Consortium (Aus-ROC) OHCA Epistry, which houses data from the individual cardiac arrest registries of EMS across Australia and New Zealand [2, 7]. Each EMS provides data for all attended OHCAs (OHCA where an ambulance attended the scene including those where resuscitation was not attempted) with the exception of two Australian regions, NT and ACT, who only provide data for cases where resuscitation was attempted by EMS. The Epistry contains detailed information concerning patient demographics, arrest characteristics, pre-EMS interventions (including bystander-administered CPR), and OHCA survival which are collected in accordance with Utstein definitions [8]. Data are subject to rigorous quality control and ongoing review to ensure standardisation. The data for this project were extracted from the Epistry on the 2^nd^ June, 2022.

### Inclusion criteria

Patients aged 20 years or older with OHCAs of medical aetiology were included in the study [8]. This age range was chosen to align with the Australian Bureau of Statistics census data [9].

### Local government areas and geospatial mapping

Every state and territory (except the ACT) has a separate system of local government, with LGAs administered by a council (or equivalent) which makes decisions on local, town or city matters. In Australia, parts of the country that are not administered by an incorporated local government body are identified as an Unincorporated area; the ACT, an unincorporated area, is represented by a single spatial unit.

For LGAs, we obtained the 2017 LGA boundary file (Environmental Systems Research Institute shapefile) from the Australian Bureau of Statistics [10]. The S2 Fig shows a national map of LGA boundaries, of which there were 543. We based the spatial location of each arrest on ambulance dispatch coordinates (latitude and longitude). We then linked each arrest to its corresponding LGA, using a point-in-polygon spatial intersection. All maps and spatial operations were performed in ArcMap version 10.7.

### Calculation of OHCA incidence and bystander CPR rates

The incidence of EMS-attended OHCAs of medical aetiology in adults aged 20 years or older was calculated for each LGA using 2017 population data as the denominator. Bystander CPR rates amongst bystander-witnessed arrests were calculated. The restriction to bystander-witnessed arrests was to avoid potential confounding of regional variation in witnessed OHCAs [5].

Due to the absence of data on untreated OHCA cases from NT and the ACT, the expected number of untreated OHCAs was estimated by applying the average rate for untreated OHCAs across the remaining six EMS. Since 43.8% of attended OHCAs received an EMS resuscitation attempt across those jurisdictions, the estimated number of untreated cases in NT and ACT was calculated as OHCA (NT/ACT) / 0.438. Due to the univariate nature of our analyses, we decided this simple method of mean ratio imputation [11] was the most parsimonious approach to scaling the numerators for these two territories. The same mean ratio approach was used to estimate the number of bystander-witnessed events among untreated OHCAs in the NT and ACT, along with the corresponding counts for bystander CPR.

All calculations were performed at the LGA level within NT and for Unincorporated ACT.

### Bayesian spatial analysis

Bayesian spatial models were used to obtain smoothed estimates of OHCA incidence and bystander CPR rates. We adapted a model that we developed previously to investigate spatial heterogeneity in Queensland in: (1) OHCA incidence and (2) bystander CPR rates [6]. The model was run separately for each state/territory with LGA being the spatial cells.

The Bayesian analysis is described in detail in the S1 File. The observed number of cases occurring in LGA *i*, *X*_i_, was modelled following a zero-inflated Poisson (ZIP) distribution with mean *E*_i_*μ*_i_. ZIP was used due to the presence of zero counts in our data. The model takes the following form:

$$X_{i}∼ZIP\left(E_{i}\mu_{i}\right)$$ (Eq 1)

In Eq 1 above, *E*_i_ is the population of LGA *i*, which acts as an offset. *μ*_i_ represents the incidence rate, which can be expressed as follows:

log(OHCAincidenceinaspecificLGA)=(intercept)+(structuredspatial)+(unstructuredspatial) (Eq 2)

where the intercept represents the average incidence in the state, the structured component models spatially-correlated heterogeneity in incidence, and the unstructured component models spatially-uncorrelated heterogeneity.

Bystander CPR rate for each LGA, *π*_i_, was modelled by using the number of bystander CPR cases for that LGA *Y*_i_ as the response variable and the number of bystander-witnessed arrests *w*_i_ as the offset. *Y*_i_ was modelled following a zero-inflated Binomial (ZIB) distribution as follows:

$$Y_{i}∼ZIB\left(\pi_{i},w_{i}\right)$$ (Eq 3)

The spatial random effects were specified on the logistic transformation of *π*_i_ as follows:

logit(proportionofcasesreceivingbystanderCPRinaspecificLGA)=(intercept)+(structuredspatial)+(unstructuredspatial) (Eq 4)

In Eq 4 above, the intercept and the spatial components have the same interpretations as in Eq 2, but with regard to bystander CPR rates.

Model parameters were inferred using the integrated nested Laplace approximation (INLA) method. INLA was implemented in the INLA package for the R programming language (the *R-INLA* package). Like our previous study [6], we used noninformative prior distributions on the parameters and hyperparameters. For the precision hyperparameters, we used Penalised Complexity prior with parameters *U* = 1 and *α* = 0.01 as motivated by Riebler et al. [12] and Peluso et al. [13]. For the mixing parameter, we used Penalised Complexity prior with parameters *U* = 0.5 and *α* = 2/3 according to Riebler et al. [12]. The prior on the intercepts was a uniform distribution; and the prior on the coefficients was a Gaussian distribution *N*(0, 0.001) as per the default specification of the INLA [14].

We validated the model by comparing the differences between model estimated and observed numbers of events. The majority of the dots fall onto the line of equality, indicating an excellent agreement between model estimates and observations (S3 Fig).

We also identified high-risk LGAs nationally and for each state/territory. At the national level, high-risk LGAs were defined as those having an incidence rate higher than the national 75^th^ percentile and a bystander CPR rate less than the national 25^th^ percentile. For each state and territory, high-risk LGAs were those with incidence rate greater than the state or territory’s 75^th^ percentile and bystander CPR rate less than the state or territory’s 25^th^ percentile. For visualisation purposes, maps of the identified high-risk LGAs for each state and territory were overlaid with shapefile of the state/territory remoteness map. Areas within each state/territory of Australia are classified as metropolitan, regional or remote according to the Australian Statistical Geographic Standard (ASGS) Remoteness Structure 2016 [10]. All analyses were performed in R (version 3.6.1).

### Missing data

Data for witnessed status was missing in 5% of arrests (n = 3426). These were included in the estimation of incidence but were excluded from the analysis of bystander CPR rates. Of the OHCA with missing data on witnessed status, 88% died at scene. In the Aus-ROC Epistry, OHCAs leading to an on-scene death are predominantly unwitnessed arrests. Given that the analysis of bystander CPR is restricted to bystander-witnessed arrests, the exclusion of these missing cases is unlikely to have had a substantial impact on the estimation of bystander CPR rates. Bystander CPR was missing amongst 2% of the bystander-witnessed arrests (n = 389). These were also excluded from the analysis of bystander CPR rates but retained in the estimation of OHCA incidence.

### Ethics approval

Ethics approval was independently sought by each of the contributing registries. Overarching ethics approval for the Aus-ROC Epistry was provided by the Monash University HREC (MUHREC; Project ID: 13933). The MUHREC granted a waiver of patient consent under The Health Records Act 2001 (Victoria, Australia).

De-identified data are held by the Aus-ROC Epistry, but for quality assurance purposes, the data are re-identifiable by each ambulance service. Re-identifiable is defined by the NHMRC, as “data from which identifiers have been removed and replaced by a code, but it remains possible to re-identify a specific individual by, for example, using the code or linking different data sets.” Re-identification can only be undertaken by the data custodians of the individual registries.

## Results

### Description of data

A total of 62,579 EMS-attended, adult OHCA cases of medical aetiology were recorded between January 2017 to December 2019. These were distributed across 543 LGAs nationwide. As expected, data on event counts show a spatial concentration of cases in LGAs with high population density situated on the east coast of the country, reflecting the patterns of population distribution in Australia (S4 Fig). There were 15,970 bystander-witnessed arrests, of which, 10,534 received bystander CPR.

### Bayesian analysis

**Fig 1:**
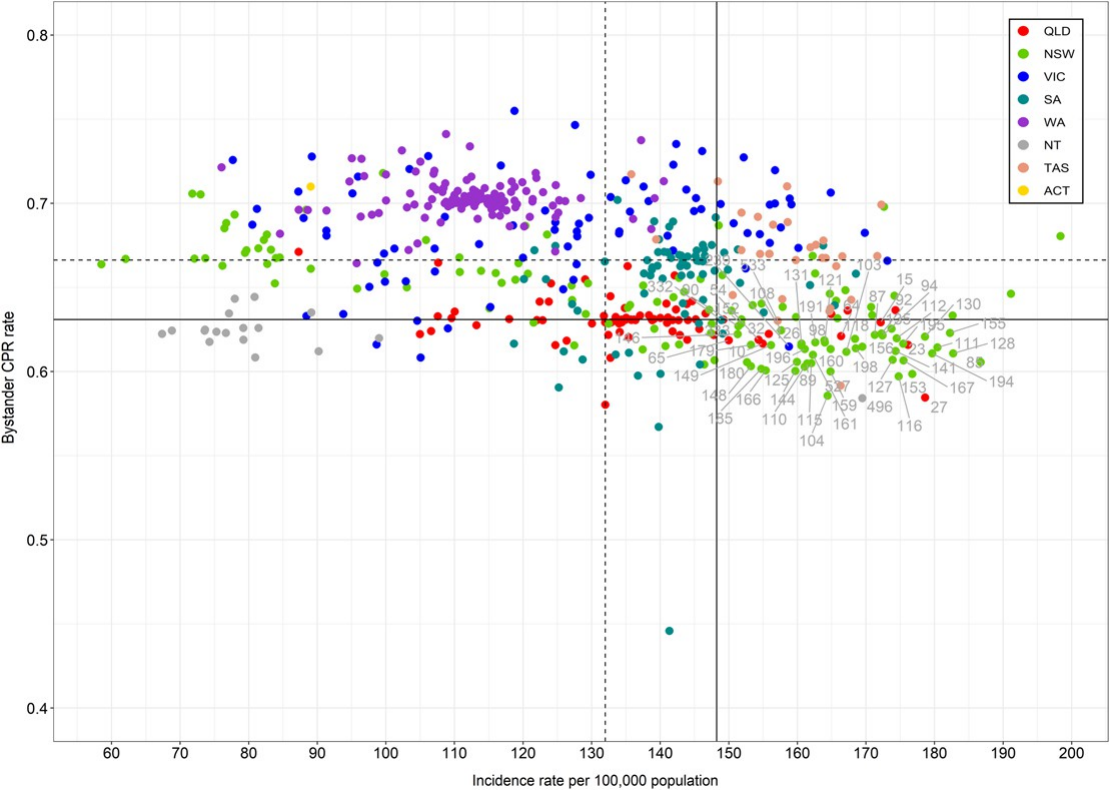
National high-risk areas, defined as incidence rate > national 75^th^ percentile (vertical continuous line) and rate of bystander cardiopulmonary resuscitation (CPR) < national 25^th^ percentile (horizontal continuous line). Dotted lines represent the medians for incidence rate (vertical line) and bystander CPR (horizontal line).

The scatterplot in Fig 1 shows the national distribution of LGAs according to bystander CPR and OHCA incidence rates. Estimated incidence ranged from 58.5 (North Sydney LGA, NSW) to 198.3 (Mid Coast LGA, NSW); bystander CPR rates ranged from 0.45 (Mount Gambier LGA, SA) to 0.75 (Macedon LGA, Victoria). In total, 60 LGAs fell in the high-risk quadrant. These were predominantly located in regional and remote LGAs in NSW (n = 46).

**Fig 2:**
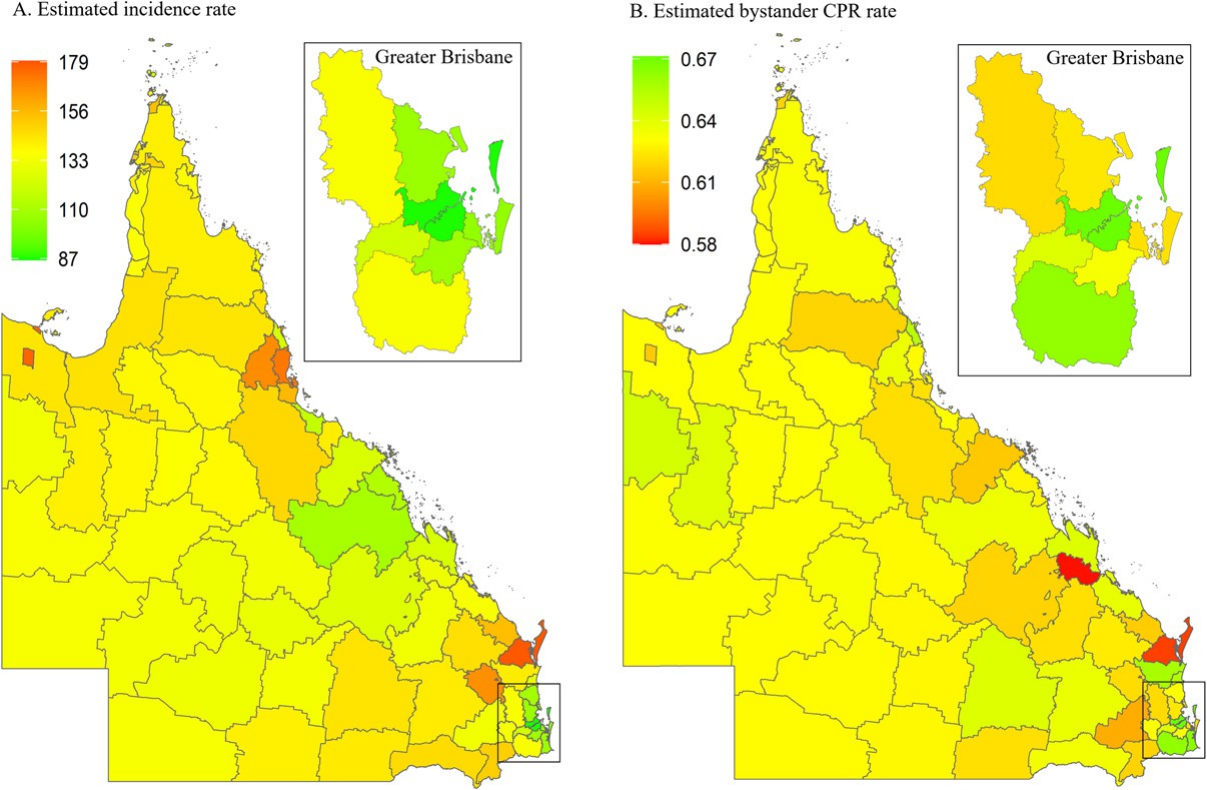
Posterior mean of estimated incidence rate (per 100,000 population per year) and of estimated bystander CPR rate for each LGA in Queensland. CPR, cardiopulmonary resuscitation; LGA, local government area.

**Fig 3:**
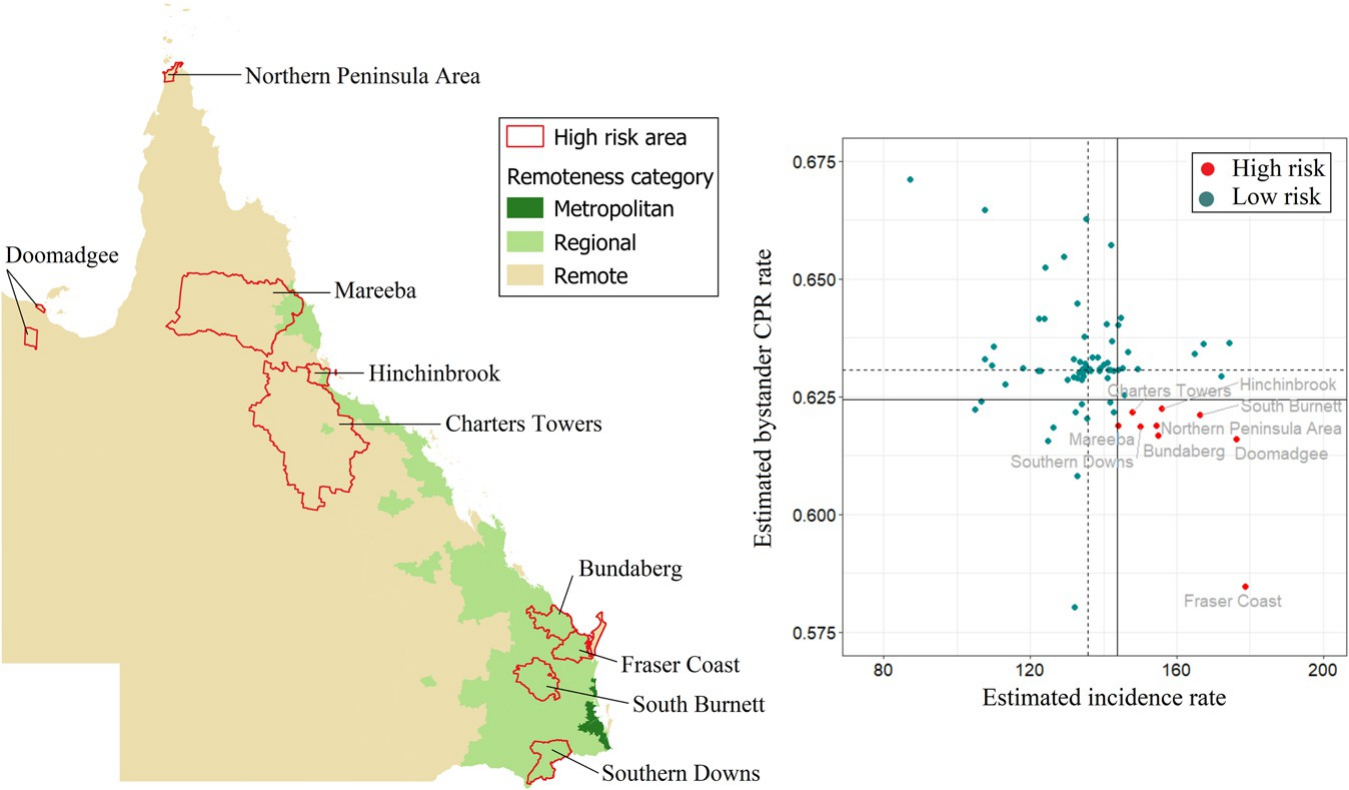
Queensland high-risk areas, defined as incidence rate > state 75^th^ percentile (vertical continuous line) and rate of bystander cardiopulmonary resuscitation (CPR) < state 25^th^ percentile (horizontal continuous line). Dotted lines represent the medians for incidence rate (vertical line) and bystander CPR (horizontal line).

Fig 2 thematically visualises the estimated mean incidence rate and the estimated bystander CPR rate for each LGA in the state of Queensland. The S1 File, shows numerical values for each LGA. Estimated incidence rates range from 87 (Brisbane LGA) to 179 (Fraser Coast LGA) cases per 100,000 population per year. Overall, OHCA incidence is highest in the northern and southern parts of the state as well as two south-eastern LGAs. OHCA incidence is lowest in Greater Brisbane area. The greater capital city area also has the highest bystander CPR rates. Nine LGAs in Queensland were identified as high-risk areas (Fig 3). All these LGAs are classified as regional or remote (Fig 3).

**Fig 4:**
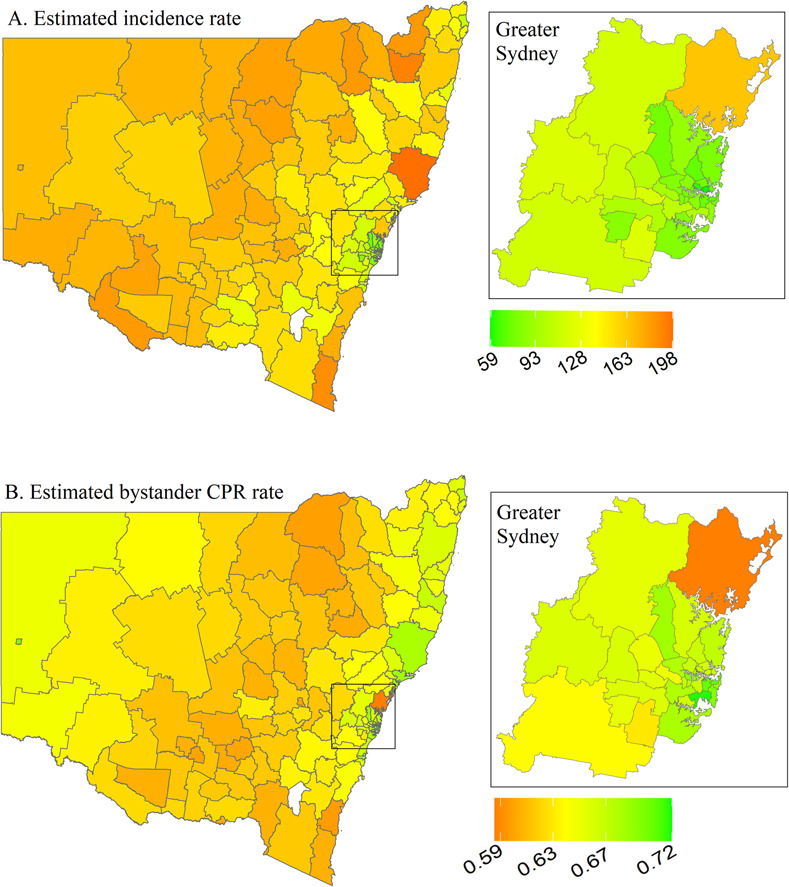
Posterior mean of estimated incidence rate (per 100,000 population per year) and of estimated bystander CPR rate for each LGA in New South Wales. CPR, cardiopulmonary resuscitation; LGA, local government area.

**Fig 5:**
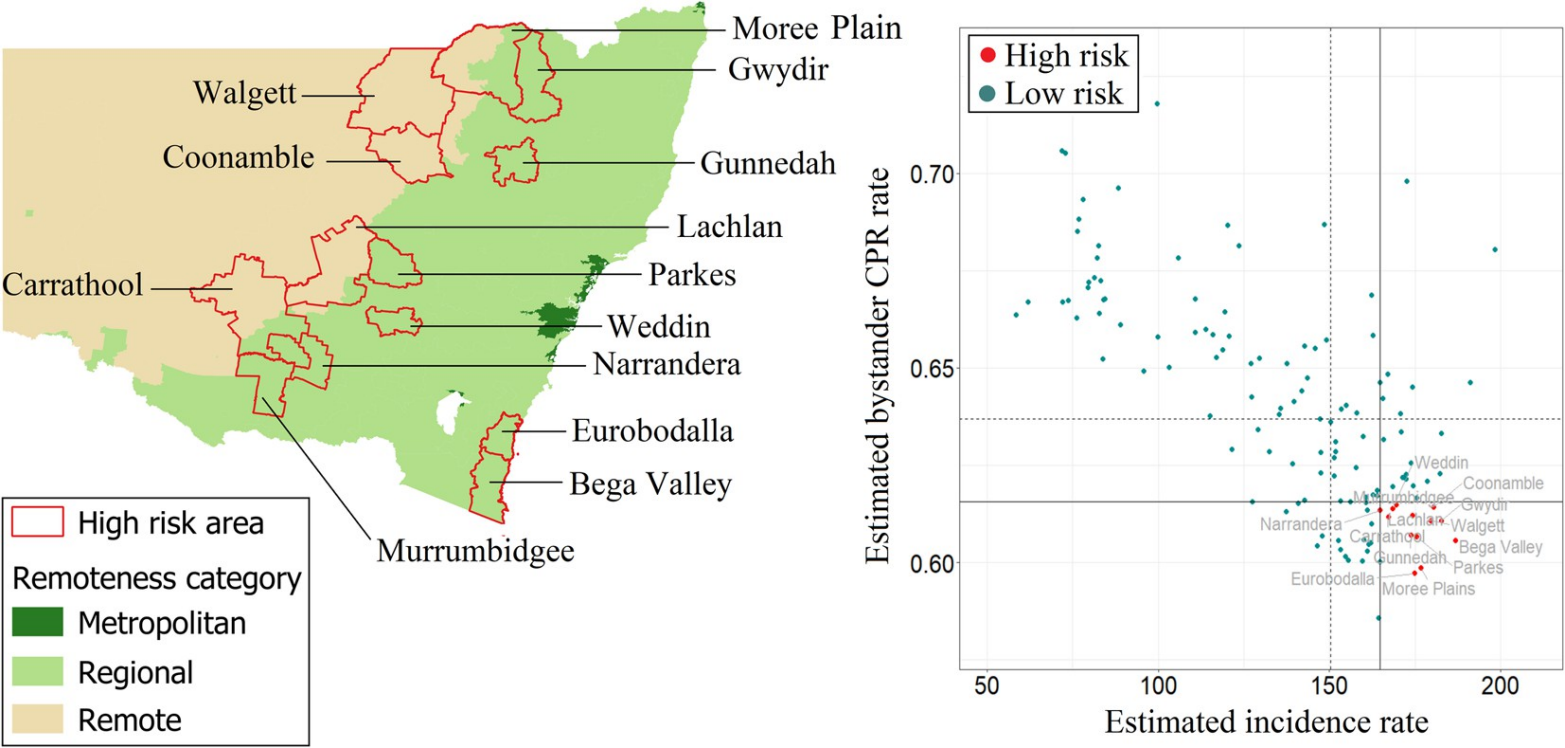
New South Wales high-risk areas, defined as incidence rate > state 75^th^ percentile (vertical continuous line) and rate of bystander cardiopulmonary resuscitation (CPR) < state 25^th^ percentile (horizontal continuous line). Dotted lines represent the medians for incidence rate (vertical line) and bystander CPR (horizontal line).

Estimated incidence rate and bystander CPR rate for NSW are visualised in Fig 4, with LGA-specific numerical values shown in the S1 File. Incidence rates ranged from 58 to 198 cases per 100,000, with the lowest rates found along the east coast. The east coast of the state also had the highest bystander CPR rates. Most high-risk areas are located in central NSW and are classified as regional/remote (Fig 5). There appears to be an inverse relationship between incidence and bystander CPR rates such that bystander CPR rates decrease as incidence increases (Fig 5).

**Fig 6:**
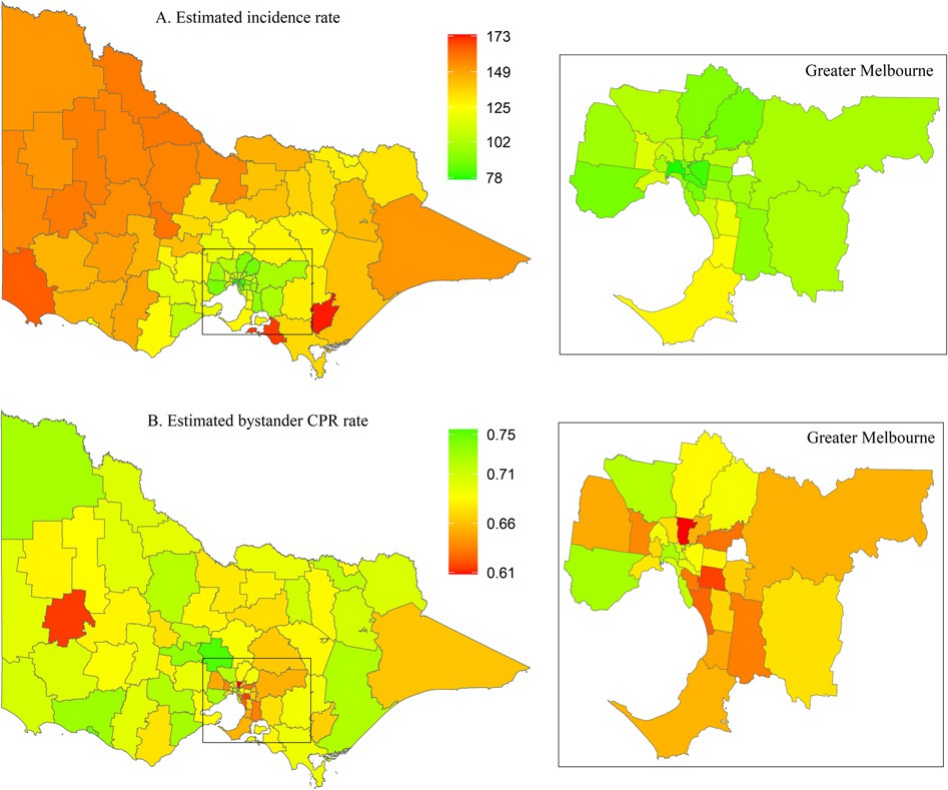
Posterior mean of estimated incidence rate (per 100,000 population per year) and of estimated bystander CPR rate for each LGA in Victoria. CPR, cardiopulmonary resuscitation; LGA, local government area.

**Fig 7:**
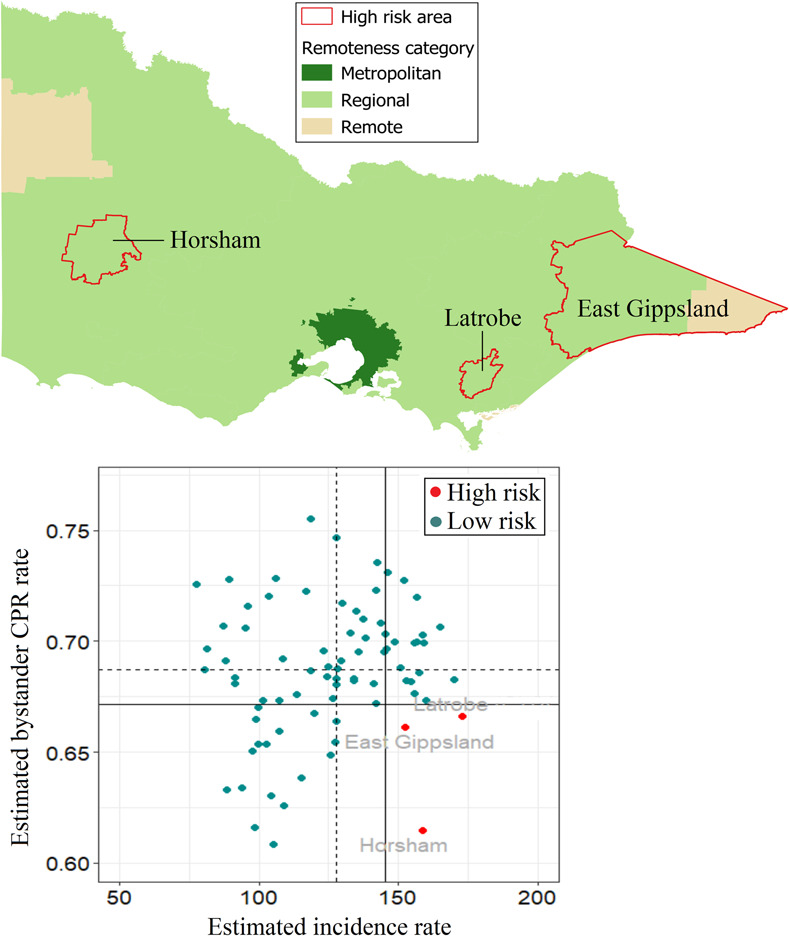
Victoria high-risk areas, defined as incidence rate > state 75^th^ percentile (vertical continuous line) and rate of bystander cardiopulmonary resuscitation (CPR) < state 25^th^ percentile (horizontal continuous line). Dotted lines represent the medians for incidence rate (vertical line) and bystander CPR (horizontal line).

For Victoria, OHCA incidence ranged from 80 to 173 cases per 100,000 and is highest in the western areas of the state and lowest in the greater capital city (Fig 6). The rates of bystander CPR are fairly consistent across the LGAs, with a few exceptions of relatively lower bystander CPR rates (Fig 6). Three areas are identified as high-risk areas. Like other states, these areas are classified as regional/remote areas (Fig 7). LGA-specific numerical values can be found in the S1 File.

**Fig 8:**
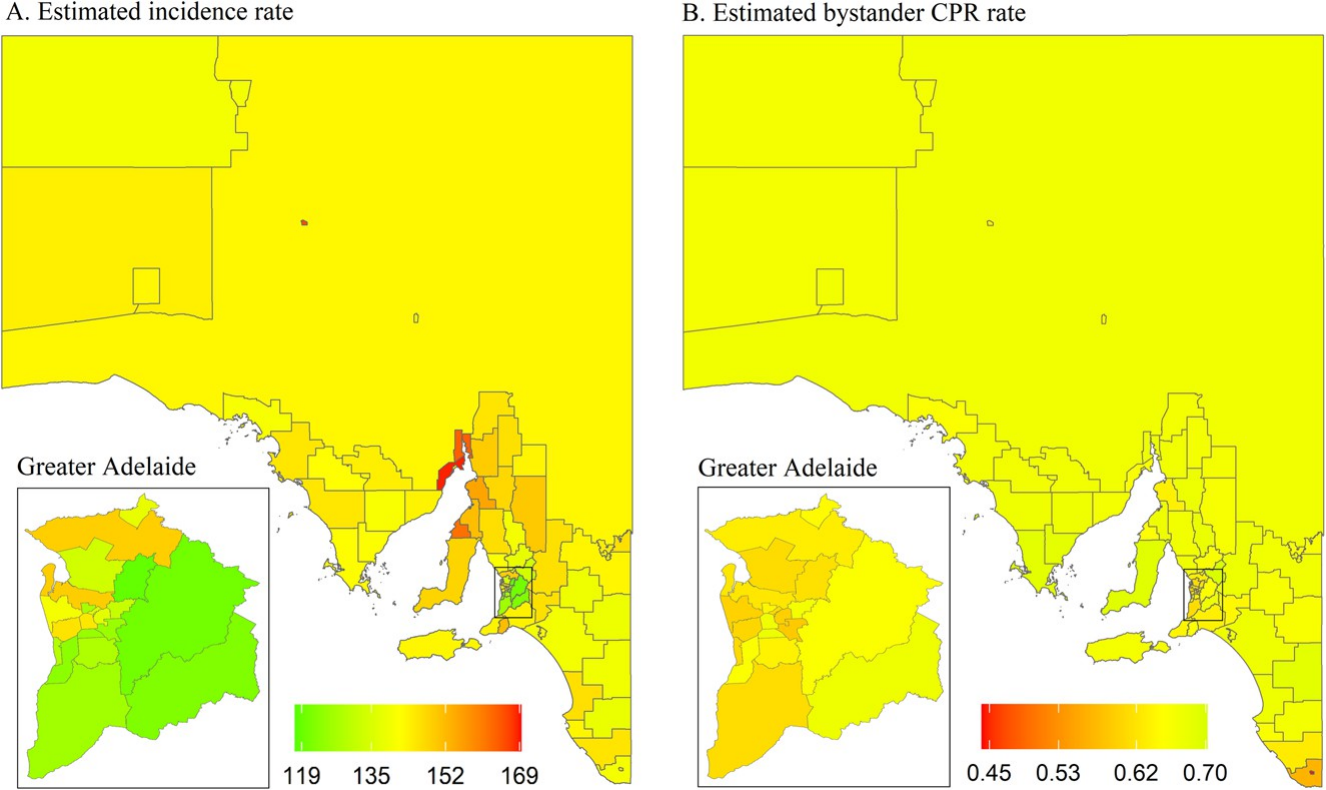
Posterior mean of estimated incidence rate (per 100,000 population per year) and of estimated bystander CPR rate for each LGA in South Australia. CPR, cardiopulmonary resuscitation; LGA, local government area.

**Fig 9:**
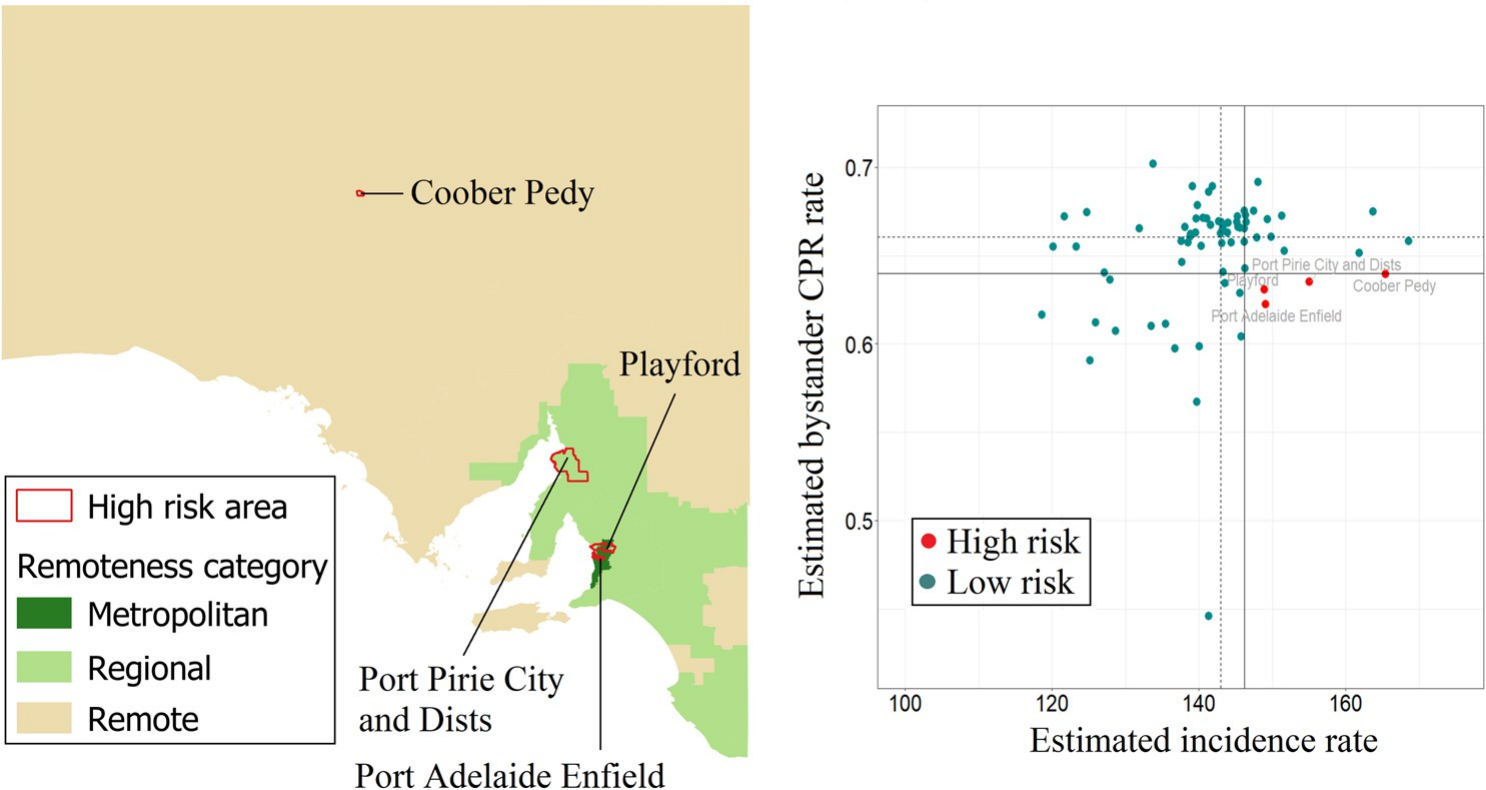
South Australia high-risk areas, defined as incidence rate > state 75^th^ percentile (vertical continuous line) and rate of bystander cardiopulmonary resuscitation (CPR) < state 25^th^ percentile (horizontal continuous line). Dotted lines represent the medians for incidence rate (vertical line) and bystander CPR (horizontal line).

Estimated OHCA incidence in SA ranged from 119 to 169 cases per 100,000 population, with Greater Adelaide having the lowest incidence rate (Fig 8). Estimated bystander CPR rates appear to be fairly consistent across the state (Fig 8). Four LGAs were deemed high-risk, two of which are metropolitan areas (Fig 9). LGA-specific numerical values can be found in the S1 File.

**Fig 10:**
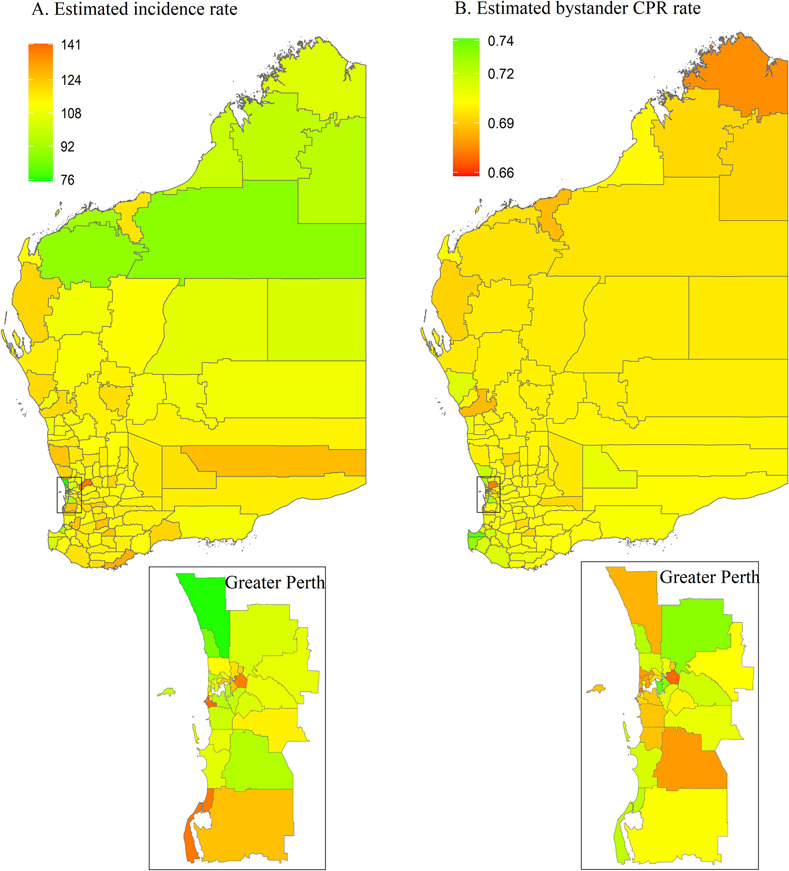
Posterior mean of estimated incidence rate (per 100,000 population per year) and of estimated bystander CPR rate for each LGA in Western Australia. CPR, cardiopulmonary resuscitation; LGA, local government area.

**Fig 11:**
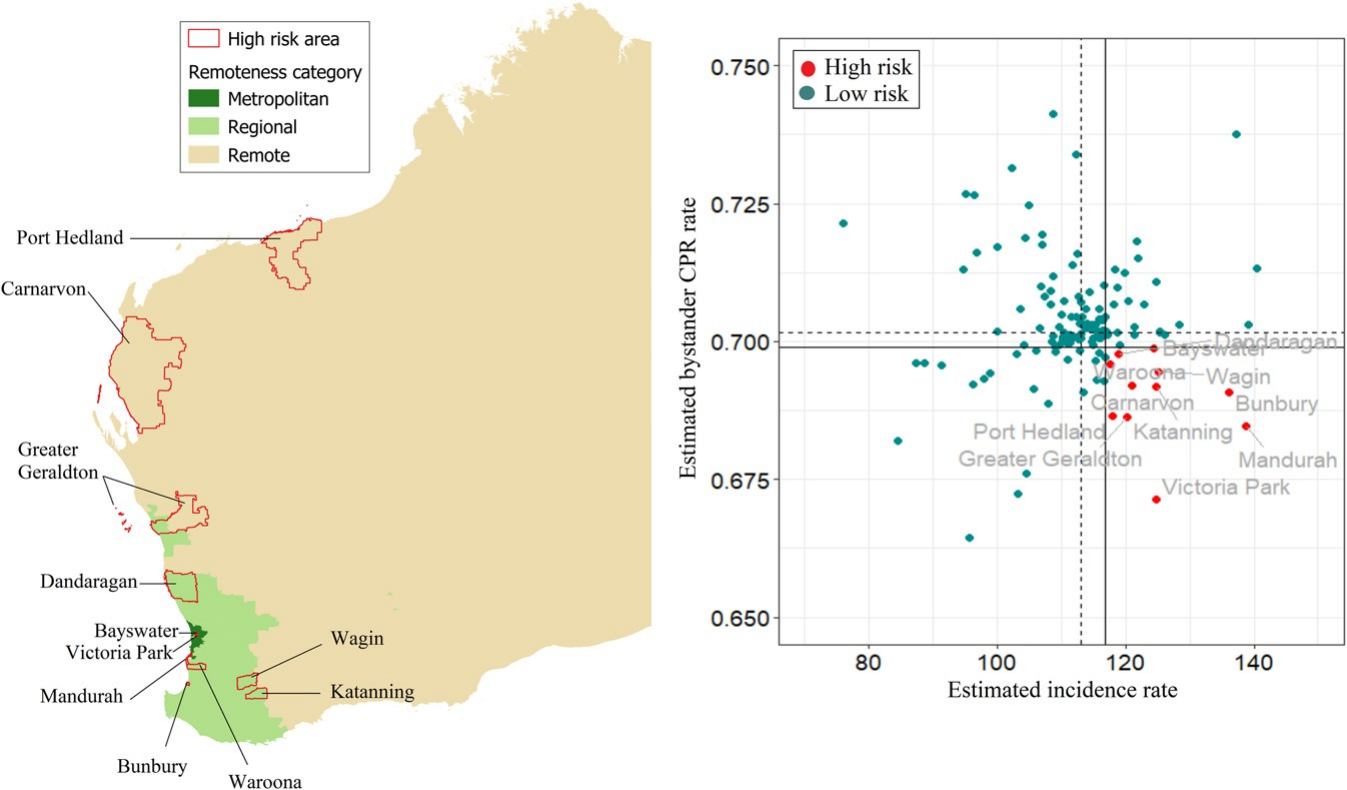
Western Australia high-risk areas, defined as incidence rate > state 75^th^ percentile (vertical continuous line) and rate of bystander cardiopulmonary resuscitation (CPR) < state 25^th^ percentile (horizontal continuous line). Dotted lines represent the medians for incidence rate (vertical line) and bystander CPR (horizontal line).

Fig 10 shows estimated incidence and bystander CPR rates for WA, with numerical values shown in the S1 File. OHCA incidence ranges from 76 to 140 cases per 100,000 and is highest in the southern and south-western parts of the state. Bystander CPR rates are highest in the south-western part of the state. There are 11 high-risk areas, scattering from central west to south-west of WA (Fig 11), two of which were located in metropolitan areas.

**Fig 12:**
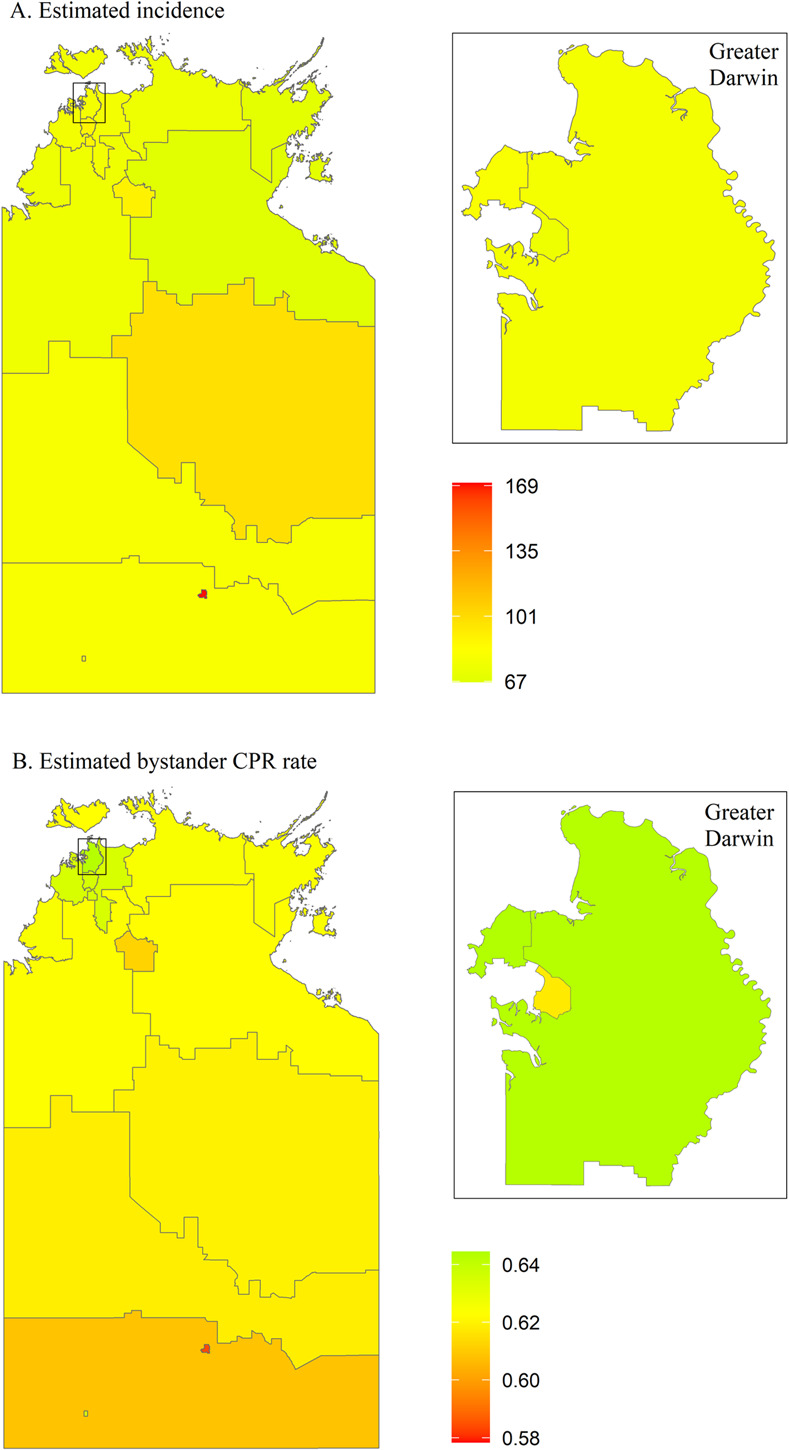
Posterior mean of estimated incidence rate (per 100,000 population per year) and of estimated bystander CPR rate for each LGA in Northern Territory. CPR, cardiopulmonary resuscitation; LGA, local government area.

**Fig 13:**
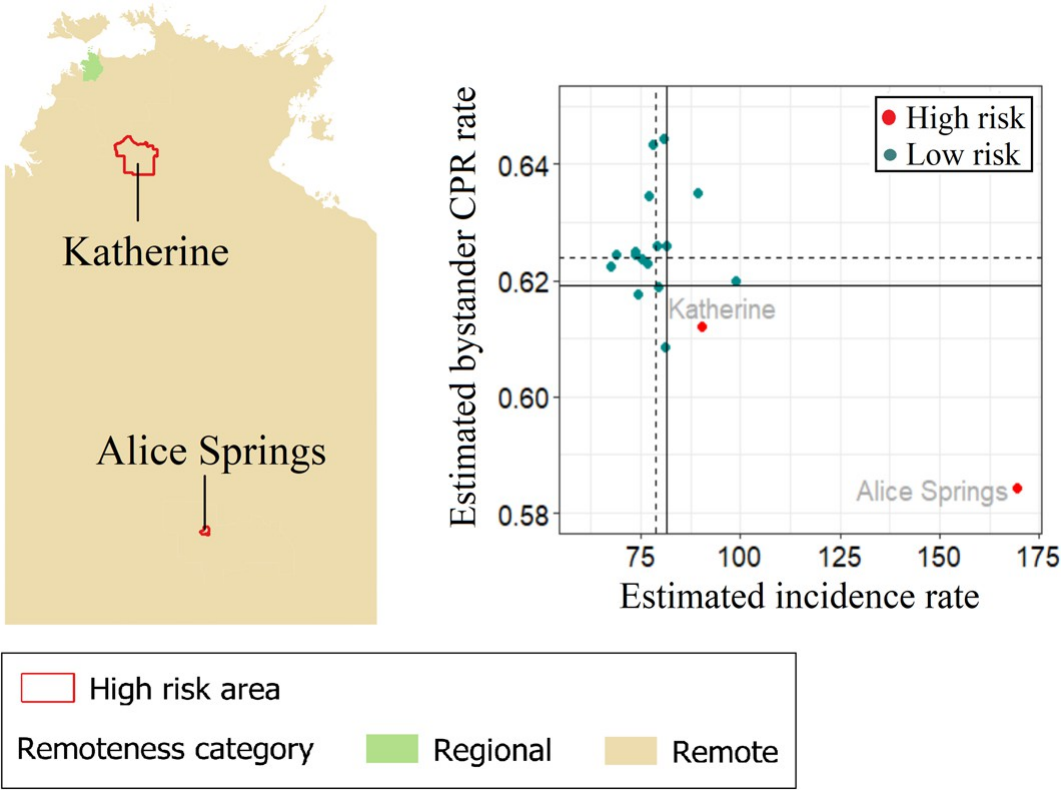
Northern Territory high-risk areas, defined as incidence rate > state 75^th^ percentile (vertical continuous line) and rate of bystander cardiopulmonary resuscitation (CPR) < state 25^th^ percentile (horizontal continuous line). Dotted lines represent the medians for incidence rate (vertical line) and bystander CPR (horizontal line).

OHCA incidence in NT ranges from 67 to 169 cases per 100,000 and is fairly similar across the state, with a few exceptions where the incidence is relatively high (Fig 12). Alice Springs has the highest incidence and lowest bystander CPR rate. Alice Springs and Katherine are high-risk areas, both of which are categorised as remote areas (Fig 13). LGA-specific numerical values can be found in the S1 File.

**Fig 14:**
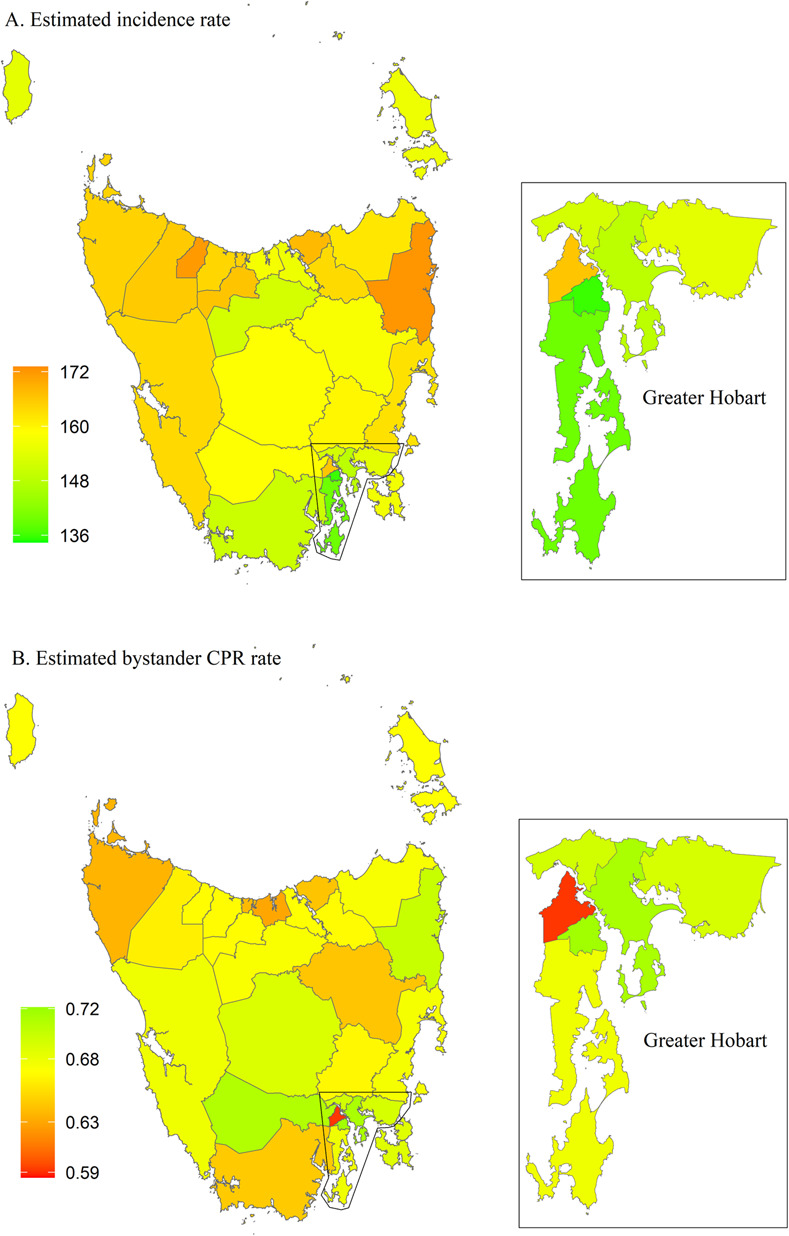
Posterior mean of estimated incidence rate (per 100,000 population per year) and of estimated bystander CPR rate for each LGA in Tasmania. **CPR,** cardiopulmonary resuscitation; LGA, local government area.

**Fig 15:**
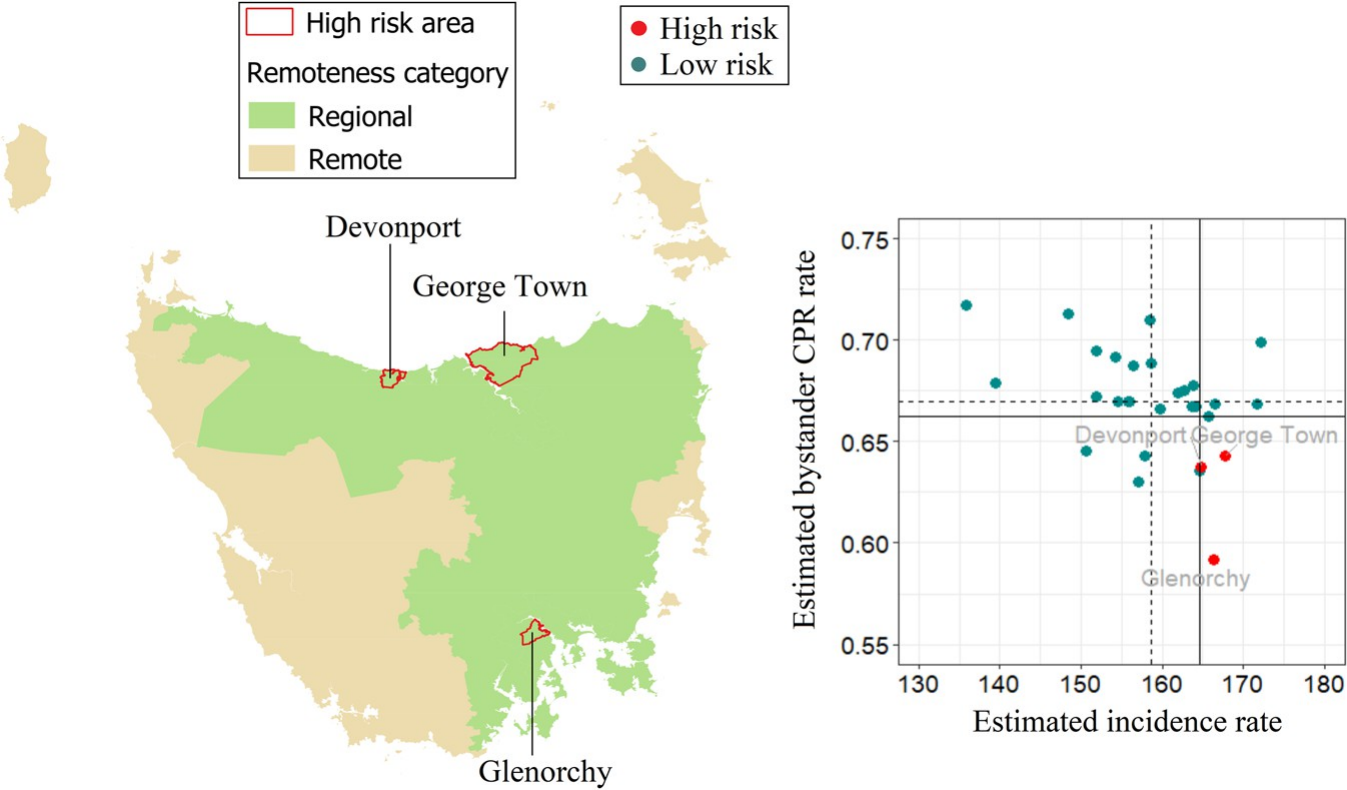
Tasmania high-risk areas, defined as incidence rate > state 75^th^ percentile (vertical continuous line) and rate of bystander cardiopulmonary resuscitation (CPR) < state 25^th^ percentile (horizontal continuous line). Dotted lines represent the medians for incidence rate (vertical line) and bystander CPR (horizontal line).

For Tasmania, the incidence was high and ranged from 135 to 172 cases per 100,000 persons and is highest in the north-eastern and north-western part of the state, and lowest in Greater Hobart (Fig 14). Three areas are considered high-risk, including Devonport, George Town and Glenorchy (Fig 15). LGA-specific numerical values can be found in the S1 File.

## Discussion

While Bayesian spatial analysis has previously been used to study regional variations in OHCA incidence across Queensland and Victoria [5, 6, 15], this is the first study to apply these methods across all Australian states and territories. Regional variation in both OHCA incidence and bystander CPR rates were observed in all states and territories. As with the earlier Victorian study [5, 15], high-risk areas with an increased incidence of OHCA and low bystander CPR rates were able to be identified. These have a total population of 1.17 million adults and were predominantly located outside of major metropolitan areas.

Previous studies have focused on describing the underlying population characteristics of high-risk regions as an adjunct to the development of targeted interventions [15–19]. This includes the characterisation of regions according to the demographic (population size and density) and socioeconomic (area education and employment levels, ethnicity, area disadvantage) characteristics, as well as area prevalence of health risk factors. A Victorian paper reported that area characteristics such as the proportion of population over 65 years, socioeconomic status, smoking prevalence and education level were significant predictors of OHCA incidence in a multivariable model and explained 93.9% of the variation in OHCA incidence across LGA [15]. Another Victorian paper has reported LGAs with low rates of bystander CPR (bystander witnessed) have low rates of CPR training [20]. These factors are likely to account for much of the variation across LGAs at the national level.

Our identification of regions at highest risk across Australia provides areas where interventions may be best targeted. Based on our maps, high-risk LGAs are typically located in the regional and remote areas of the nation. National data indicates that the prevalence of self-reported heart, stroke and vascular disease is similar across remoteness areas [21]; however, mortality from coronary heart disease (CHD) is higher in remote and very remote areas when compared to Australia overall [21]. Age is strongly linked to cardiovascular morbidity and mortality. This varies across regions with the proportion of persons aged above 65 years being higher in regional communities compared to major cities [21]. However, age is not likely to provide a full account for our findings as the proportion of residents aged above 65 years is lowest in remote and very remote areas where many of our high-risk regions are located. The prevalence of health-risk behaviours such as tobacco use and alcohol consumption at risk levels is also higher in regional and remote Australia when compared to that in major cities [21]. Public health campaigns targeting health-risk behaviour may reduce the prevalence of CHD and its associated mortality in rural and remote regions. However, this is more likely to produce long-term results than immediate gains with respect to reducing OHCA incidence in those areas.

Local data has shown that large, expensive mass media campaigns directed at early recognition of cardiac warning signs can lead to substantial reductions in the incidence of OHCA and associated mortality rates [22]. These appear to work by increasing awareness of heart attack symptoms and addressing the known barriers associated with calling EMS early (e.g. ignoring or downplaying symptoms and embarrassment) [23, 24]. Campaign awareness also appears to influence actions in response to cardiac symptoms and is associated with an increased likelihood of seeking medical attention [24] and presenting to hospital in the first hours after symptom onset [25]. However, this campaign was only effective at reducing OHCA incidence while it was active and for a short period of time after [22]. Similarly, symptom awareness and recognition was not maintained once exposure to advertising ceased [23]. This indicates that media campaigns must be ongoing, or at least repeated regularly, for maximum benefit. However, national campaigns are expensive, and the cost of ongoing campaigns may be prohibitive. Targeting specific, high-risk LGAs through their local media (television and radio networks and local print media) and according to local characteristics may reduce overall costs and provide better reach into regional and remote communities, thus providing a more cost-effective approach [23].

Improving bystander CPR rates nationally may also be aided by targeting regions with low rates. Surveys of Australian adults have shown low awareness of the need for CPR training and where access to training is limited [26]. Improving awareness and access to training in high risk regions may be a better use of resources. A recent review has found CPR training outcomes can be achieved by self-directed learning, such as video/DVD self-instruction [27]. Self-directed learning may provide a better option to traditional CPR training in rural and remote regions and can be delivered at a lower cost. The school setting provides an additional option for training in CPR. Available evidence suggests that school-based training enhances adolescents’ knowledge, skills and confidence in providing CPR and pupils trained in CPR can serve as CPR multipliers by passing the acquired skills and knowledge onto family members and friends [28]. School catchment areas in rural and remote regions typically cover geographically dispersed populations, thereby potentially extending reach to adults in more remote regions. A pilot study is currently underway examining school-based CPR training in Victoria, Australia [29]. In person grass-roots CPR education, such as the Heart Safe Community (HSC) project that has been implemented in regional areas across Victoria [30], show great promise in building community capacity to respond to OHCA, and may explain the changes in LGAs CPR rates between our study and previous research in this region [5]. The project has also demonstrated the effectiveness of community leadership in advocating for improved access to automated external defibrillators (AEDs). A community-based cluster randomised trial (FirstCPR) of CPR and AED training targeting regions with high rates of migrant populations is currently underway in NSW [31, 32].

Placing volunteer first responder programs in high-risk regions may be an effective strategy for improving OHCA survival rates. The odds of survival to hospital discharge are up to 3-fold higher in OHCA cases attended by volunteers [33], which may in part reflect higher rates of CPR and AED use prior to EMS arrival [34]. The success of such programs is dependent on having volunteers available, who are near the OHCA event and are willing and able to attend. This may be a limiting factor in remote regions characterised by smaller populations dispersed over a large geographical area. It has been reported that at least ten volunteer first responders and two public access AED per km^2^ are needed for an optimum response [35]. However, the implementation and maintenance costs are modest [33], they can be easily integrated into a broader community approach and may be effective in larger regional centres and towns.

### Limitations

The Aus-ROC Epistry collects data from all EMS servicing Australia with a near-complete capture of OHCAs occurring across the nation. There may be regional differences in case definitions, however, the collection is subject to ongoing review and any differences are likely to be minor. Two ambulance services only provide data for EMS-treated OHCAs and incidence had to be estimated using the rates for EMS resuscitation attempts in other services. These are the two smallest EMS in Australia and the extrapolation is unlikely to affect the broader picture.

Data were missing for witnessed status (5% of arrests) and bystander CPR (2%), creating the potential for biased estimates of bystander CPR rates. We decided not to undertake a sensitivity analyses around these missing data due to the highly complex state space in our dataset (543 LGAs). The choice of LGA as the spatial unit also has some limitations. LGAs typically encompass multiple suburbs and localities and as a result, can be quite heterogeneous with respect to OHCA incidence and the socioeconomic factors that drive it. However, Australia consists of large tracts of sparsely populated land and results based on finer granulations may be unreliable in those areas. Furthermore, LGAs provide an administrative structure that facilitates localised intervention.

We also acknowledge that there is a lack of consistency about how “high-risk” regions should be defined and we have adopted the cut-offs used by Sasson et al. [36]. We have placed tables of numerical values of incidence and bystander CPR in the S1 File so that readers and policy-makers can explore different cut-offs.

### Future directions

These data serve as a starting point for future studies of regional variations in OHCA outcomes and associated modifiable factors. We are currently investigating regional variations in survival and plan to add a temporal component to map changes in regional variations over time. This would support evaluations of interventions targeted at reducing regional disparities in OHCA incidence and survival and also allow us to develop models to predict OHCA risk over time [37]. A grant application for a simulation study examining the effect of manipulating modifiable factors on OHCA incidence and survival is currently under review. We also believe that, given the high survival seen with public AED use [38], research is needed to map AED locations in our region and explore the association with survival.

## Conclusions

This study highlights the value of this type of analysis internationally in that it shows how this resource can be used to direct intervention to regions where it is needed most. We have observed considerable regional disparities throughout Australia and pinpointed specific LGAs that pose a high risk. These areas exhibit a combination of high incidence rates of cardiac arrest and low rates of bystander CPR. Interestingly, these LGAs are predominantly situated in the remote and regional parts of Australia. To effectively address this issue, it is crucial to implement strategies that focus on community engagement and target regions identified as having the greatest risk. By doing so, we can ultimately reduce the occurrence of OHCAs and improve the current low rates of survival.

## Supporting information

S1 Fig Australia map with state/territory boundaries and the boundaries of the capital city of each state/territory. (TIF)

S2 Fig Australia map with Local Government Area boundaries. (TIF)

S3 Fig Comparison of model estimated and observed number of events. (TIF)

S4 Fig Population density (20+ year-old persons per km2) (top) and number of paramedic attended out-of-hospital cardiac arrest (bottom). (TIF)

S1 File Bayesian spatial analysis. (DOCX)

S1 Checklist STROBE statement—checklist of items that should be included in reports of observational studies. (DOCX)
