# Correction: Identifying areas of Australia with high out-of-hospital cardiac arrest incidence and low bystander cardiopulmonary resuscitation rates: A retrospective, observational study

**DOI:** 10.1371/journal.pone.0303681

**Published:** 2024-05-09

**Authors:** Tan Doan, Stuart Howell, Stephen Ball, Judith Finn, Peter Cameron, Emma Bosley, Bridget Dicker, Steven Faddy, Ziad Nehme, Natalie Heriot, Andy Swain, Melanie Thorrowgood, Andrew Thomas, Samuel Perillo, Mike McDermott, Tony Smith, Karen Smith, Jason Belcher, Janet Bray, on behalf of the Aus-ROC OHCA Epistry Management Committee

In Fig 10, one of the smaller maps (bystander CPR rates Greater Perth) was incorrectly coloured. Please see the correct Fig 10 here.

**Fig 10:**
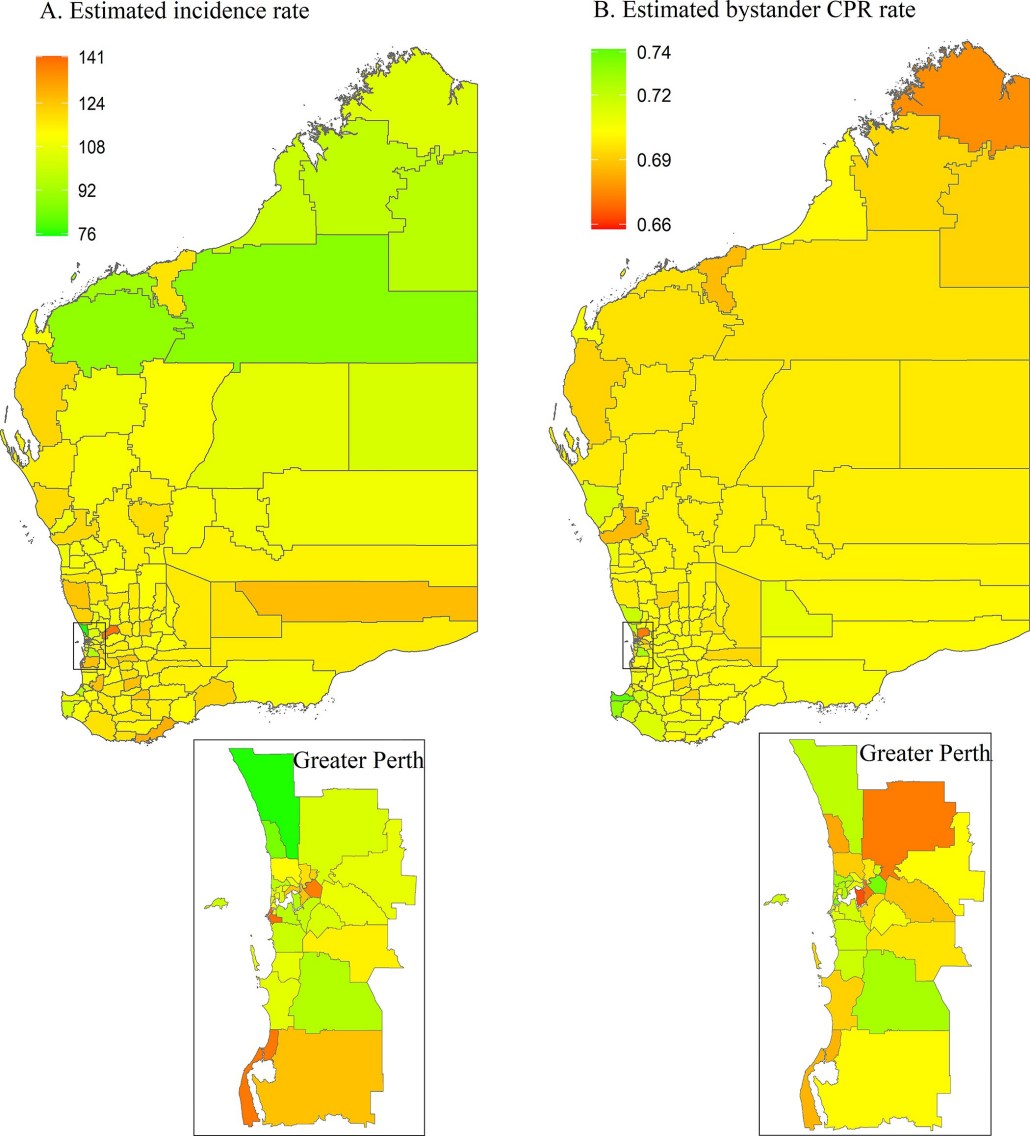
Posterior mean of estimated incidence rate (per 100,000 population per year) and of estimated bystander CPR rate for each LGA in Western Australia. CPR, cardiopulmonary resuscitation; LGA, local government area.
