# Supplementary figures and images for: Identifying areas of Australia with high out-of-hospital cardiac arrest incidence and low bystander cardiopulmonary resuscitation rates: A retrospective, observational study
Source: PLoS One. 2024 Apr 23;19(4):e0301176. doi: 10.1371/journal.pone.0301176 (PMC11037527; doi:10.1371/journal.pone.0301176)

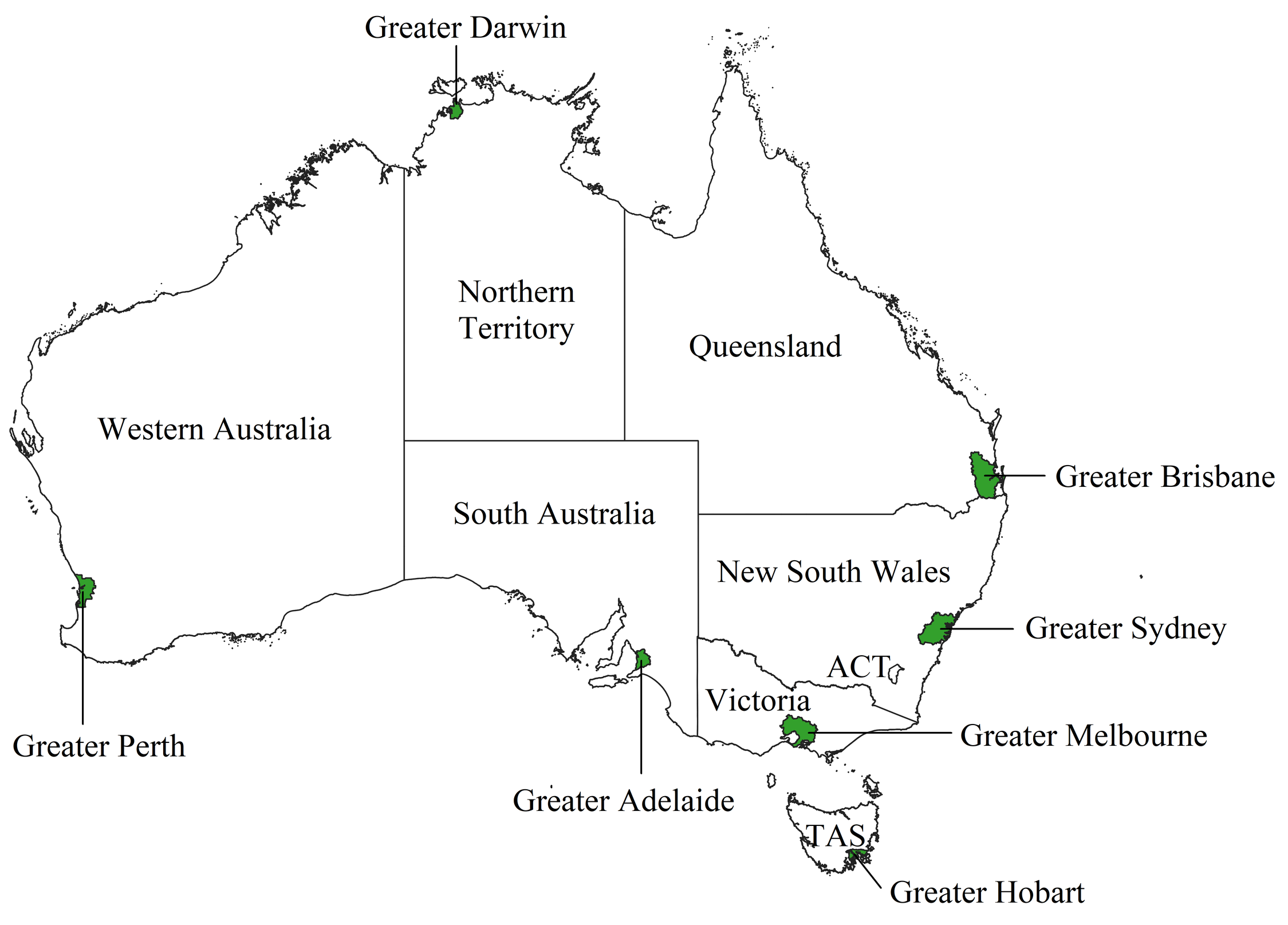

Supplement: S1 Fig — (TIF) [file pone.0301176.s001.tif]

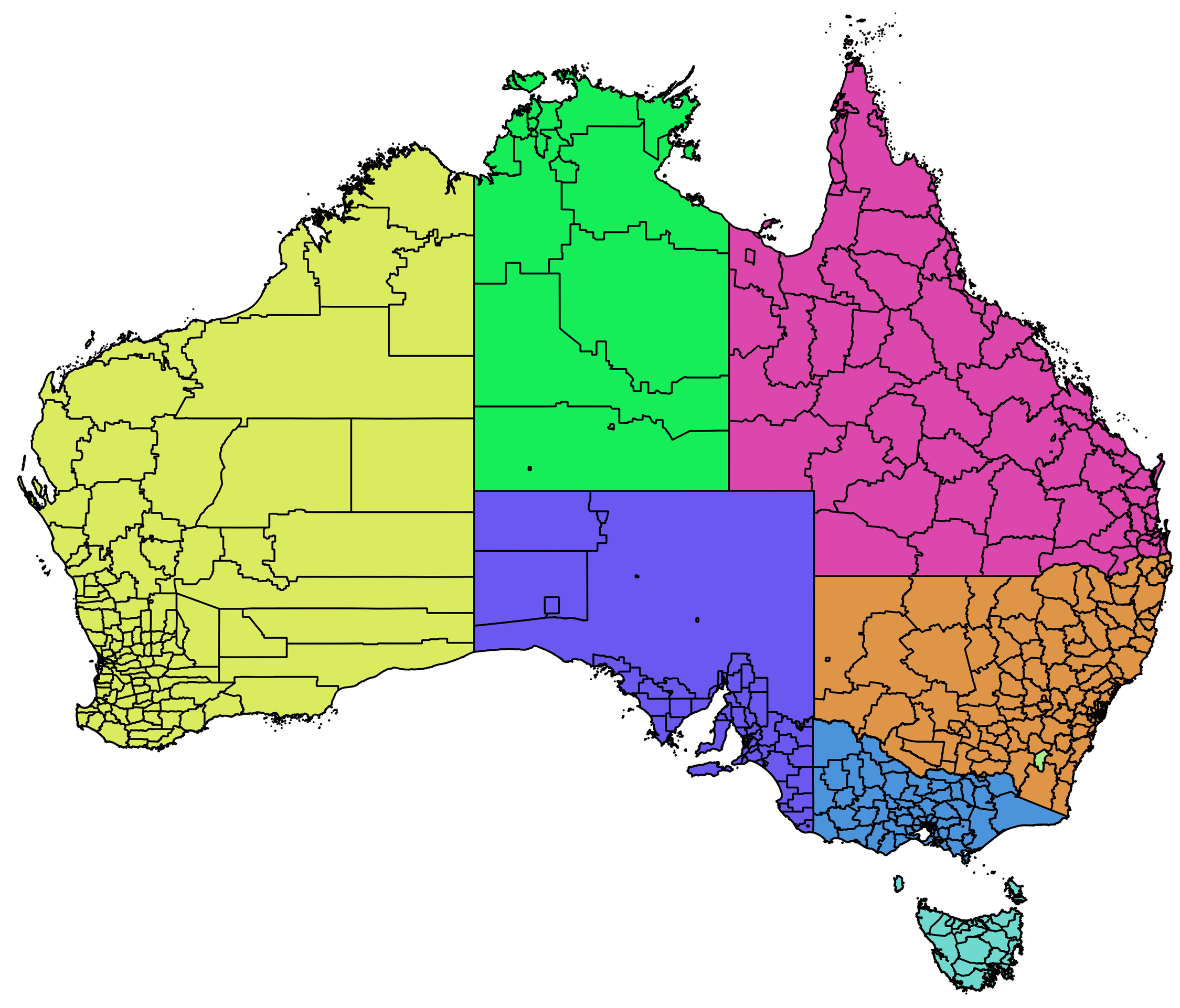

Supplement: S2 Fig — (TIF) [file pone.0301176.s002.tif]

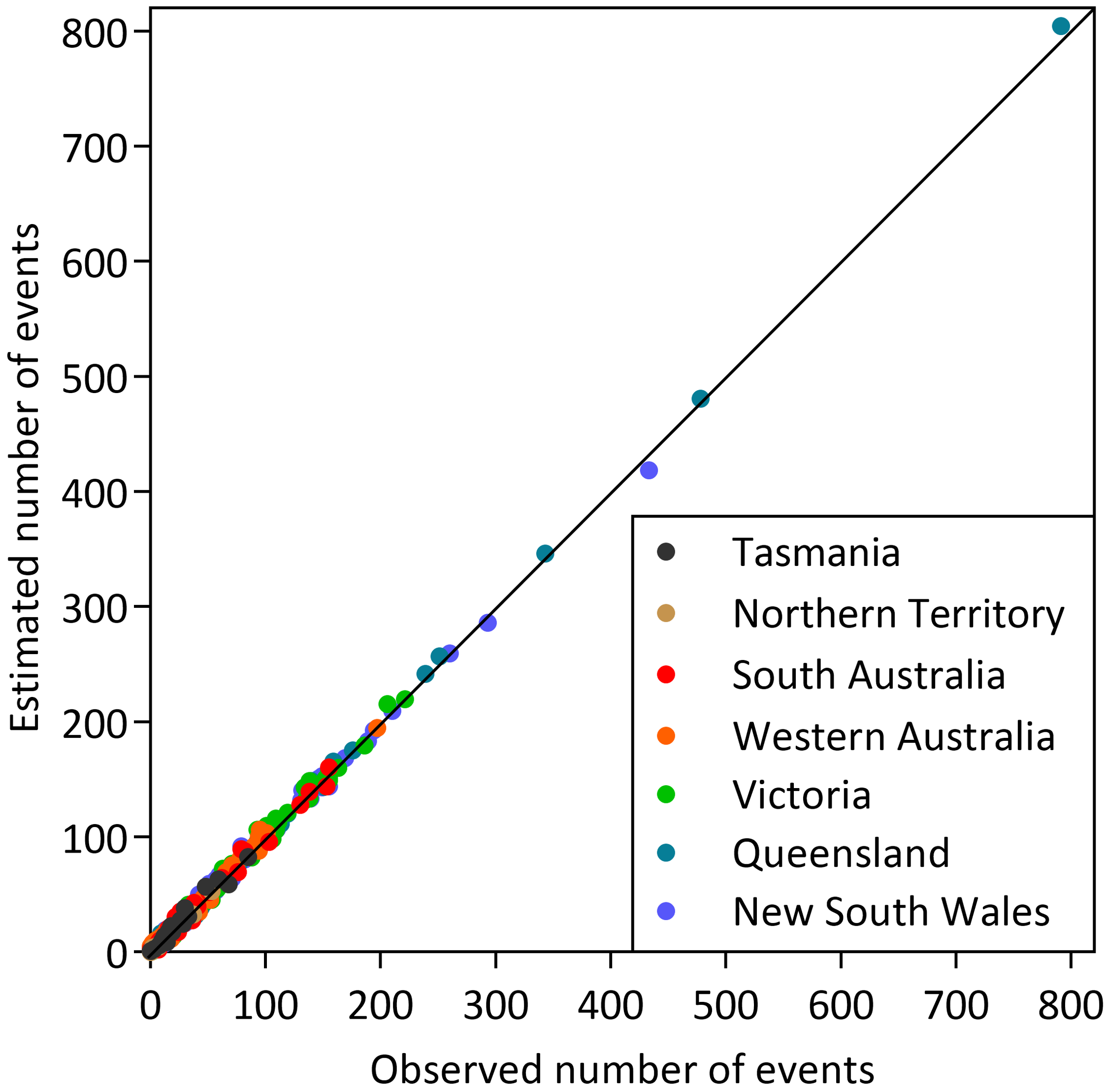

Supplement: S3 Fig — (TIF) [file pone.0301176.s003.tif]

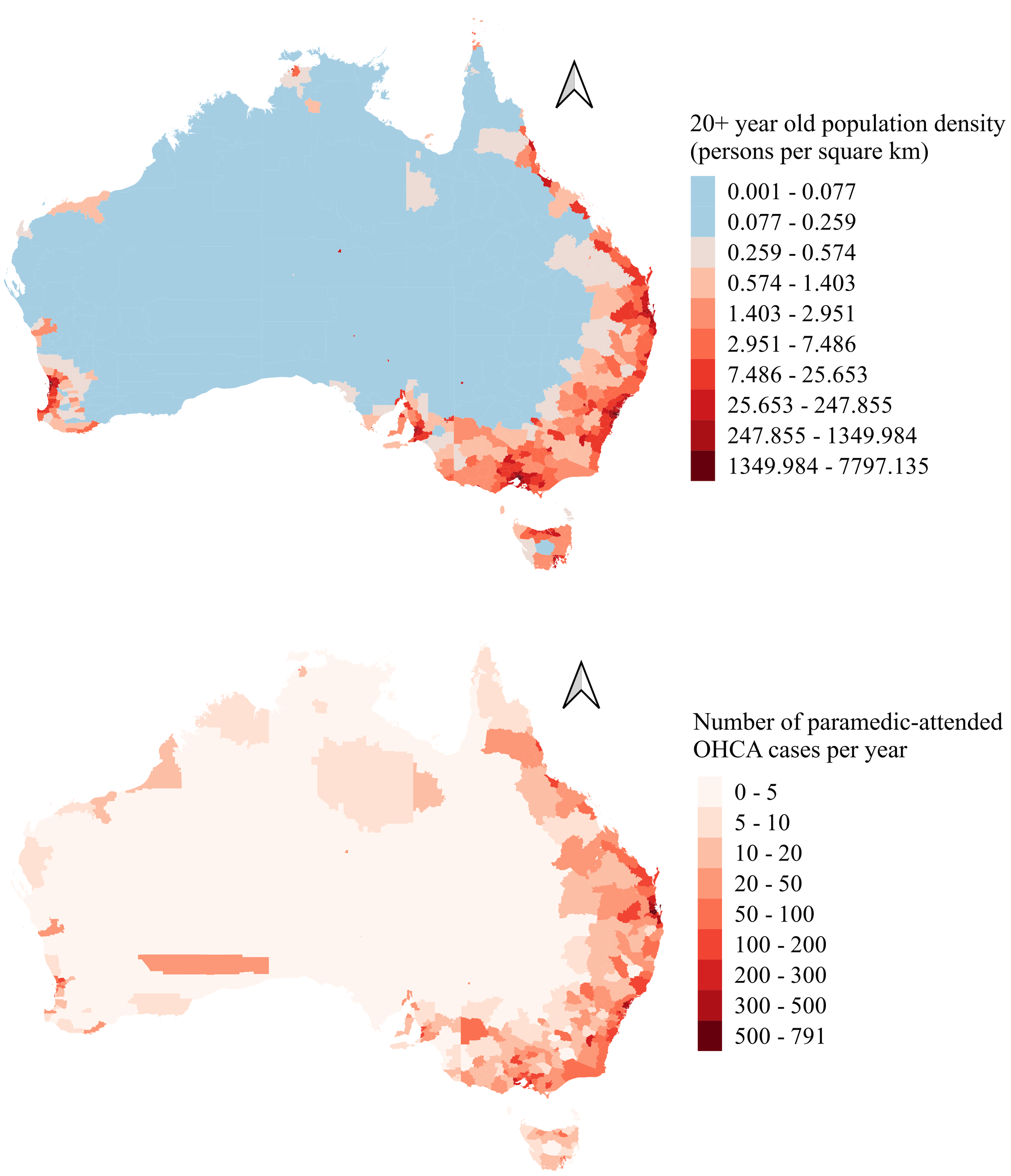

Supplement: S4 Fig — (TIF) [file pone.0301176.s004.tif]
