# Supplementary material for: Identifying areas of Australia with high out-of-hospital cardiac arrest incidence and low bystander cardiopulmonary resuscitation rates: A retrospective, observational study
Source: PLoS One. 2024 Apr 23;19(4):e0301176. doi: 10.1371/journal.pone.0301176 (PMC11037527; doi:10.1371/journal.pone.0301176)
Supplement: S1 File — (DOCX) [file pone.0301176.s005.docx]

**Bayesian spatial analysis**

We adapted the model that we developed previously to investigate spatial heterogeneity in (1) OHCA incidence and (2) bystander CPR rates for each state/territory with LGA being the spatial cells.[1]

The observed number of cases occurring in LGA *i*, *X*_i_, was modelled following a zero-inflated Poisson (ZIP) distribution with mean $E_{i}\mu_{i}$. ZIP was used due to the presence of zero counts in our data. The model takes the following form:

$$X_{i}\sim ZIP\left( E_{i}\mu_{i} \right) (\mathrm{eq}. 1)$$

In equation 1 above, *E_i_* is the population of LGA *i*, which acts as an offset. *μ_i_* represents the incidence rate. *μ_i_* can be expressed as follows:

$\log\left( \mu_{i} \right)= \mu+u_{i}+v_{i}$ (eq. 2)

where *μ* represents the overall incidence in the state; *u_i_* is the spatially structured residual, and *𝜐_i_* is the unstructured residual.

Bystander CPR rate for each LGA, *π*_i_, was modelled by using the number of bystander CPR cases for that LGA *Y*_i_ as the response variable and the number of bystander-witnessed arrests *w*_i_ as the offset. *Y*_i_ was modelled following a zero-inflated Binomial (ZIB) distribution as follows:

$Y_{i}\sim ZIB\left( \pi_{i}, w_{i} \right)$ (eq. 3)

The spatial random effects were specified on the logistic transformation of *π*_i_ as follows:

$\mathrm{logit} \left( \pi_{i} \right)= \mu+u_{i}+v_{i}$ (eq. 4)

In equation 4 above, the intercept and the spatial components have the same interpretations as in equation 2, but with regard to bystander CPR rates.

As per our previous study,[1] a reparameterised version of the Besag, York and Mollie model (called BYM2) was used for the spatial components of both the models for incidence and for bystander CPR rates. The parameters and hyperparameters of the models can be conveniently inferred using the integrated nested Laplace approximation (INLA) method. We used noninformative prior distributions on the parameters and hyperparameters. For the precision hyperparameters, we used Penalised Complexity prior with parameters *U* = 1 and *α* = 0.01 as motivated by Riebler et al.[2] and Peluso et al.[3] For the mixing parameter, we used Penalised Complexity prior with parameters *U* = 0.5 and *α* = 2/3 according to Riebler et al.[2] The prior on the intercepts was a uniform distribution; and the prior on the coefficients was a Gaussian distribution *N*(0, 0.001) as per the default specification of the INLA.

We also identified high risk LGAs for each state/territory, defined as LGAs with incidence rate greater than the state 75^th^ percentile and bystander CPR rate less than the state 25^th^ percentile. All analysis was performed in R (version 3.6.1).

**References**

1. Doan TN, Wilson D, Rashford S, Ball S, Bosley E. Spatiotemporal variation in the risk of out-of-hospital cardiac arrests in Queensland, Australia. Resusc Plus. 2021;8:100166.

2. Riebler A, Sorbye SH, Simpson D, Rue H. An intuitive Bayesian spatial model for disease mapping that accounts for scaling. Stat Methods Med Res. 2016;25(4):1145-65.

3. Peluso S, Mira A, Rue H, Tierney NJ, Benvenuti C, Cianella R, et al. A Bayesian spatiotemporal statistical analysis of out-of-hospital cardiac arrests. Biom J. 2020;62(4):1105-19.
