# Supplementary material for: Identifying areas of Australia with high out-of-hospital cardiac arrest incidence and low bystander cardiopulmonary resuscitation rates: A retrospective, observational study
Source: PLoS One. 2024 Apr 23;19(4):e0301176. doi: 10.1371/journal.pone.0301176 (PMC11037527; doi:10.1371/journal.pone.0301176)
Supplement: S1 Checklist — (DOCX) [file pone.0301176.s006.docx]

STROBE Statement—checklist of items that should be included in reports of observational studies

|  | Item No. | Recommendation | Page  No. | Relevant text from manuscript |
| --- | --- | --- | --- | --- |
| **Title and abstract** | 1 | (*a*) Indicate the study’s design with a commonly used term in the title or the abstract | **Page 1** | **a retrospective, observational study.** |
|  |  | (*b*) Provide in the abstract an informative and balanced summary of what was done and what was found | **Page 2** |  |
| Introduction | | | |  |
| Background/rationale | 2 | Explain the scientific background and rationale for the investigation being reported | **Page 3** |  |
| Objectives | 3 | State specific objectives, including any prespecified hypotheses | **Page 3** |  |
| Methods | | | |  |
| Study design | 4 | Present key elements of study design early in the paper | **Pages 4-8** | see **METHOD** |
| Setting | 5 | Describe the setting, locations, and relevant dates, including periods of recruitment, exposure, follow-up, and data collection | **Page 4** | See Sections:  **Setting**  **Data source** |
| Participants | 6 | (*a*) *Cohort study*—Give the eligibility criteria, and the sources and methods of selection of participants. Describe methods of follow-up  *Case-control study*—Give the eligibility criteria, and the sources and methods of case ascertainment and control selection. Give the rationale for the choice of cases and controls  *Cross-sectional study*—Give the eligibility criteria, and the sources and methods of selection of participants | **PAGE 4** | See sections:  **Data source**  **Inclusion Criteria** |
|  |  | (*b*) *Cohort study*—For matched studies, give matching criteria and number of exposed and unexposed  *Case-control study*—For matched studies, give matching criteria and the number of controls per case |  |  |
| Variables | 7 | Clearly define all outcomes, exposures, predictors, potential confounders, and effect modifiers. Give diagnostic criteria, if applicable | **PAGE 4/5** | See Sections:  **Local Government Areas and Geospatial Mapping;**  **Calculation of OHCA incidence and bystander CPR rates** |
| Data sources/ measurement | 8* | For each variable of interest, give sources of data and details of methods of assessment (measurement). Describe comparability of assessment methods if there is more than one group | **PAGE 4** | See section:  **Data Source** |
| Bias | 9 | Describe any efforts to address potential sources of bias | **Not applicable** |  |
| Study size | 10 | Explain how the study size was arrived at | **Not applicable** |  |

Continued on next page

| Quantitative variables | 11 | Explain how quantitative variables were handled in the analyses. If applicable, describe which groupings were chosen and why | **PAGE 4/5**  **PAGE 5** | See sections:  **Local Government Areas and Geospatial Mapping;**  **Calculation of OHCA incidence and bystander CPR rates** |
| --- | --- | --- | --- | --- |
| Statistical methods | 12 | (*a*) Describe all statistical methods, including those used to control for confounding | **PAGE 5-7** | See sections:  **Bayesian spatial analysis** |
|  |  | (*b*) Describe any methods used to examine subgroups and interactions | **Not applicable** |  |
|  |  | (*c*) Explain how missing data were addressed | **PAGE 7** |  |
|  |  | (*d*) *Cohort study*—If applicable, explain how loss to follow-up was addressed  *Case-control study*—If applicable, explain how matching of cases and controls was addressed  *Cross-sectional study*—If applicable, describe analytical methods taking account of sampling strategy |  |  |
|  |  | (*e*) Describe any sensitivity analyses |  |  |
| Results | | | | |
| Participants | 13* | (a) Report numbers of individuals at each stage of study—eg numbers potentially eligible, examined for eligibility, confirmed eligible, included in the study, completing follow-up, and analysed | **PAGE 8** | Total number of eligible OHCA is reported along with the number of bystander-witnessed arrests and bystander-witnessed arrests receiving bystander CPR; there were no exclusions |
|  |  | (b) Give reasons for non-participation at each stage | **Not applicable** |  |
|  |  | (c) Consider use of a flow diagram | **Not applicable** |  |
| Descriptive data | 14* | (a) Give characteristics of study participants (eg demographic, clinical, social) and information on exposures and potential confounders | **Not applicable** |  |
|  |  | (b) Indicate number of participants with missing data for each variable of interest | **PAGE 7** | See heading:  **Missing data** |
|  |  | (c) *Cohort study*—Summarise follow-up time (eg, average and total amount) | **Not applicable** |  |
| Outcome data | 15* | *Cohort study*—Report numbers of outcome events or summary measures over time | **Not applicable** |  |
|  |  | *Case-control study—*Report numbers in each exposure category, or summary measures of exposure | **Not applicable** |  |
|  |  | *Cross-sectional study—*Report numbers of outcome events or summary measures | **PAGE 8** | See Section: Results |
| Main results | 16 | (*a*) Give unadjusted estimates and, if applicable, confounder-adjusted estimates and their precision (eg, 95% confidence interval). Make clear which confounders were adjusted for and why they were included | **PAGES 8-12; SUPPLEMENTARY FILE, TABLES S1-S7** |  |
|  |  | (*b*) Report category boundaries when continuous variables were categorized | **Not applicable** |  |
|  |  | (*c*) If relevant, consider translating estimates of relative risk into absolute risk for a meaningful time period | **Not applicable** |  |

Continued on next page

| Other analyses | 17 | Report other analyses done—eg analyses of subgroups and interactions, and sensitivity analyses | **Not applicable** |  |
| --- | --- | --- | --- | --- |
| Discussion | | | | |
| Key results | 18 | Summarise key results with reference to study objectives | **PAGE 13** |  |
| Limitations | 19 | Discuss limitations of the study, taking into account sources of potential bias or imprecision. Discuss both direction and magnitude of any potential bias | **PAGE 16** |  |
| Interpretation | 20 | Give a cautious overall interpretation of results considering objectives, limitations, multiplicity of analyses, results from similar studies, and other relevant evidence | **PAGES 13-16** |  |
| Generalisability | 21 | Discuss the generalisability (external validity) of the study results | **Not applicable** |  |
| Other information | |  | | |
| Funding | 22 | Give the source of funding and the role of the funders for the present study and, if applicable, for the original study on which the present article is based | **PAGE 17** |  |

*Give information separately for cases and controls in case-control studies and, if applicable, for exposed and unexposed groups in cohort and cross-sectional studies.

**Note:** An Explanation and Elaboration article discusses each checklist item and gives methodological background and published examples of transparent reporting. The STROBE checklist is best used in conjunction with this article (freely available on the Web sites of PLoS Medicine at http://www.plosmedicine.org/, Annals of Internal Medicine at http://www.annals.org/, and Epidemiology at http://www.epidem.com/). Information on the STROBE Initiative is available at www.strobe-statement.org.
